# Long-Term Clinical Outcomes of Radical Prostatectomy versus Watchful Waiting in Localized Prostate Cancer Patients: A Systematic Review and Meta-Analysis

**Published:** 2019-04

**Authors:** Mojtaba NOUHI, Seyed Masood MOUSAVI, Alireza OLYAEEMANESH, Nasser SHAKSISALIM, Ali AKBARI SARI

**Affiliations:** 1. Health Equity Research Center, Tehran University of Medical Sciences, Tehran, Iran; 2. Department of Health Service Management, School of Health Management and Information Sciences, Iran University of Medical Sciences, Tehran, Iran; 3. National Institute for Health Research, Tehran University of Medical Sciences, Tehran, Iran; 4. Department of Urology, Shahid Labbafinejad Medical Center, Shahid Beheshti University of Medical Sciences, Tehran, Iran; 5. Department of Health Management & Economics, School of Public Health, Tehran University of Medical Sciences, Tehran, Iran

**Keywords:** Clinical outcomes, Radical prostatectomy, Prostate cancer, Systematic review

## Abstract

**Background:**

The present study aimed to compare the long-term clinical and functional outcomes of patients with clinically localized prostate cancer treated with radical prostatectomy compared to the watchful waiting.

**Methods:**

PubMed, Cochrane Central Register of Controlled Trials and reference lists of relevant marker studies were scrutinized from inception to Jan 2018. Two reviewers conducted data abstraction and quality assessment of included trials independently. Quality of included studies were assessed by using Cochrane checklist. Inverse-variance and Mantel-Haenszel estimates under random effects model were used to pool results as relative risks with 95% confidence interval. Heterogeneity was assessed by using I^2^.

**Results:**

Three randomized controlled trials with 1568 participants were included. Compared to watchful waiting, radical prostatectomy had no significant effect on all-cause mortality at 12-year follow-up. However, radical prostatectomy had significant effect on reducing prostate-cause mortality at 12-year follow-up. We found significant lower prostate-cause mortality in patients with PSA>10 and GS≥7 scores who had undergone radical prostatectomy compared with patients in watchful waiting group. In addition, younger patients undergoing surgery developed lower distant metastases rate compared to another approach. Watchful waiting had a significant effect on erectile and urinary incontinence during 2 years.

**Conclusion:**

There was no significant difference between radical prostatectomy and watchful waiting on all-cause mortality. However, the radical prostatectomy was associated with statistically lower prostate-cause mortality and metastases rates. Compared with older men, younger men experienced better clinical outcomes. Moreover, watchful waiting had better effect on reducing erectile dysfunction and urinary incontinence among patients during 2 years compared to radical prostatectomy.

## Introduction

Prostate cancer is well-known as the second most diagnosed cancer of the men and the fifth most common cancer worldwide (1). Most of the developed and developing countries have also encountered increasing prevalence of the prostate cancer. Population aging (2, 3) and implementation of screening (4–7), on one hand, and some clinical characteristics of the prostate cancer such as the prolonged natural history (8, 9); on the other hand, have contributed to an increase in the number of clinically localized prostate cancer patients. As a result, these have incurred tremendous burden of the prostate cancer and its side-effects on the communities (10–13).

Hence, managing clinically localized prostate cancer to prevent unnecessary treatment, obtaining the highest benefit of the interventions and simultaneously preserving the value of the money in health care sector have become crucial issues for professionals and policy makers (14, 15). While surgery and conservative management known as two medical strategies are used for clinically localized prostate cancer patients, both have had different clinical and functional outcomes over time. Many studies, though with different methods and qualities, have attempted to represent clinical and functional outcomes of the interventions (16–21). However, recently, few well-designed studies have compared long-term outcomes of radical prostatectomy (RP) against watchful waiting (WW) and some valuable reports have published in this regard. Combining these findings with meta-analysis helps to clarify some ambiguities and answer some controversies about the long-term clinical and functional outcomes of the treatments.

## Methods

### Data sources and searches

We used comprehensive search strategies to identify reports of randomized controlled trials indexed in PubMed and The Cochrane Library from inception to Jan 2018. The language of publication was not limited to English, though. In addition, clinicaltrial.gov (https://clinicaltrials.gov) website and the reference lists of selected studies were searched to find other relevant trials. After pooling the retrieved papers and removing duplicates, two reviewers (MN & AAS) screened Title and Abstract of searched paper independently to select potentially eligible papers. Then, they read full text of the selected papers to exclude non-eligible papers and include qualified randomized controlled trials reports based on predetermined criteria. Any discrepancies raised between reviewers was discussed with third author (NS) to reach consensus.

### Study selection

We included randomized controlled studies which compared RP with WW in treatment of men who would suffer from localized prostate cancer (<T2, N0, M0). Having defined RP as procedure of removing entire prostate gland and some surrounding tissues by any procedure (e.g. perineal, laser, retropubic, laparoscopic, robotic), we also dubbed WW as any conservative approach to manage clinically localized prostate cancer which postpones initial treatment until unfavorable changes appear in clinical, pathological or biochemical features of the patients. Overall mortality was assigned as primary outcome. Other prognosis outcomes such as disease-specific mortality, local progression and metastases as well as sexual, urinary, bowel and psychological functions were assigned as the secondary outcome.

### Data extraction and quality assessment

A data abstraction form was developed and the reviewers extracted the outcomes of interest from selected studies. Any controversy between reviewers was discussed with the third author (NS) to attain consensus. General information (authors, title, journal of publication, date of publication), study population characteristics (age, race, stage, grade, prostate-specific-antigen level, gleason score, method of detection), study design details (sample size and source of funding) and study results (predefined clinical or health-related quality-of life outcomes) were, also, extracted. To assess quality of selected studies and risk of biases, the Cochrane method was used. We, subsequently, appraised randomization allocation, allocation concealment, blinding of outcome assessment, incomplete outcome data and selective report. In addition, sample size and funding source were taken into account.

### Data synthesis and analysis

We compared clinical effects of RP and WW to manage clinically localized prostate cancer. The RR was used as the principal measure to summarize clinical effect of the treatments on all outcomes. When the rates of event in participants of the study arm was available, we applied Mantel-Haenszel method. However, when the preferred information was not available, inverse-variance method was used consequently (22). To calculate pooled estimate of RR with 95% confidence interval, we applied fixed effects and random effects models. To assess heterogeneity, I^2^ test was used in which I^2^ >50% represents heterogeneity between studies. If heterogeneity of the studies was not significant, we would report the fixed effects estimation. Subgroup analyses were performed regarding age (<65≥ years). According to Cochrane method, disclosing publication bias through the Funnel plot was not reasonable for <10 included studies (22). Calculations of meta-analysis were performed by Rev.Man 5.2 Software.

## Results

**Fig. 1:**
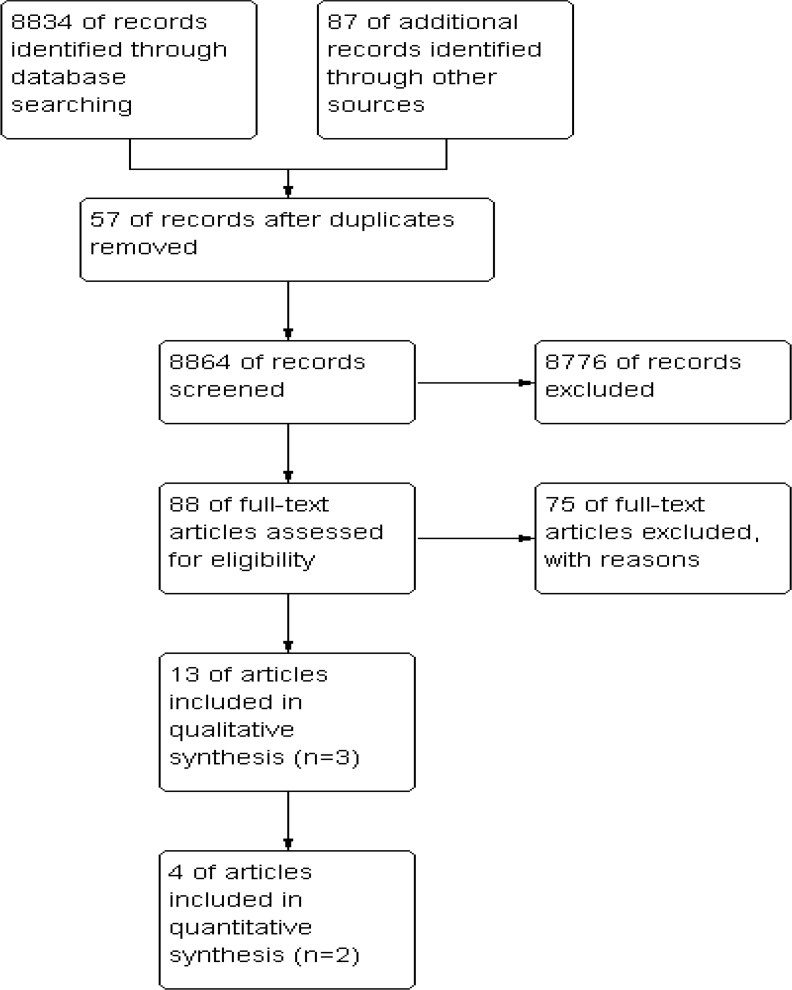
Flowchart of identification and selection of included trials

A total of 8921 studies were retrieved through database search and other previously described sources (Fig. 1).

After removing duplicates, summaries of 8864 studies were screened by the reviewers. Then, they scrutinized the full texts of the remaining 89 studies. The main reason for exclusion was non-randomized controlled studies (n=57). The remaining studies did not have either one of the predefined medical strategies (n=13) or sufficient original data (n=4). Ultimately, thirteen papers derived from three trials met inclusion criteria to be included in the study. Two of them had been conducted in United States-Prostate cancer Intervention versus Observation Trial (PIVOT) and the Veterans Administration Cooperative Urological Research Group (VACURG) - and the third in Scandinavia, Scandinavian Prostate Cancer Group-4 called (SPCG-4). Table 1 summarizes characteristics of included trials. Overall, 1568 men had participated in the included trials in which 785 of them undergone RP and remaining 783 men were allocated to WW arm. The year of reporting included trials ranged between 1981 and 2012, spanning 31 years.

**Table 1:** Characteristics of trials included in the study

***Name of trial***	***SPCG-4***	***PIVOT***	***VACURG***
Location	Scandinavian countries₮	United States	United States
Setting	14 centers	52 sites	19 hospitals
Enrollment timeframe	From October 1989 to February 1999	From November 1994 to January 2002	From May 1967 to March 1975
Participants	RP=347 WW=348	RP=364 WW=367	RP₴=74 WW₵=68
Mean age (year)	64.7	Sixty seven	RP =62.7 WW=66
Race %(Black, White, other)	NM	RP (30.5,63.7,5.9) WW(33, 60.7.1)	NM
PSA detected prostate cancer (%)	five	Seventy six (enrollees)	NM
GS distribution	RP₳(60.6, 26.2, 13.3)	WW(74.6, 24.5, 0.9)	RP(87,11, 2,)
(GS<7, GS≥7, unknown)	WW(60.9, 29.6, 9.5)	RP(71.6, 28.2, 0.2)	Placebo(86, 8, 6)
PSA distribution	RP(mean=13.5) WW(mean=12.3)	WW(mean=10.2 median=7.7) RP(mean=10.1 median=7.9)	NM
Main outcome	Prostate-cancer-mortality	All-cause mortality	Time to progression₫
Follow-up schedule	Twice a year for the first two years and then annually	Twice a year at minimum of 8 years, maximum of 15 years or patient’s death	Twice a year until 1978 and then stopped
Subgroup analysis	Age, GS, PSA	Age, race, PSA, tumor stage, tumor-risk score, Charlson score, performance score	NM
Treatment option after local progression	WW= Transurethral resection	WW= Transurethral resection₢	NM
	RP= Orchiectomy	Asymptomatic progression were discouraged	

Gleason Score/ Prostate-Specific-Antigen/NR: did not mention in trial’s reports /

₳ More than 100% refers to rounding decimal numbers which reported in original papers/

₫ includes first metastases, rising acid phosphates to twice normal or death due to the prostate cancer, then overall survival was added/

₢ some curative treatments also recommended in the protocol/

₮ it is included Sweden, Finland and Iceland countries/

₴ the strategy consists RP+ oral placebo/

₵ the strategy consists oral placebo

The VACURG, PIVOT and SPCG-4 investigators have reported their findings in four (23–26), one (27) and eight (28–35) publications. PIVOT and SPCG-4 trials compared RP with WW; while VACURG trial compared RP plus oral placebo with oral placebo alone. SPCG-4 investigators have reported all-cause death, disease-specific death, local progression and metastases of randomized participants during five (30, 32), eight (32), ten (30, 33), twelve (29) and fifteen (28) years. They, analyzed effect of age on clinical outcomes. Furthermore, functional outcomes (urinary, sexual and bowel) and psychological status were analyzed over time (31, 34, 35). PIVOT investigators reported all-cause and disease-specific mortalities and bone metastases at 4, 8 and 12 years follow-up according to race (white, black or other), age (65<years≥65), Gleason score (<7≥), PSA level (≤10>), risk of tumor (low, intermediate and high), Charlson index score (0 vs. ≥1) and self-report performance status (0 vs. 1–4) (27). VACURG investigators reported all-cause mortality after fifteen (25) and twenty three (26) years follow-up regardless of subgroup characteristics. Following the participants in all trials began similarly, twice a year, but they had experienced different follow-up plan. SPCG-4 trial underwent yearly follow-up after two years (32), VACURG trial continued until 1978 (23) and PIVOT also pursued this process for the years or patient’ death (36). PSA-detected percentage and mean age of participants relatively varied between the trials. PIVOT was a unique trial in which sufficient attention was paid to race differences (36).

### Assessment the quality of studies

Among included trials, PIVOT and SPCG-4 proved to have adequate randomization and concealment of allocation within treatments. Randomization in PIVOT and SPCG-4 was stratified based on site/center using telephone services (32, 36). SPCG-4 was also stratified based on the degree of differentiation in the grade of cancer while VACURG mentioned neither randomization process nor concealment of allocation. Regarding the nature of study arms, blinding the patients was not possible. Blinding the assessors who analyzed the outcomes to prevent selection bias was clearly reported in SPCG-4 and PIVOT trials, however, it was not reported in the VACURG trial whether they were blinded (32, 36). All trials but the VACURG used intention-to-treatment approach to analyze the outcomes. In addition, investigators in PIVOT and SPCG-4 trials reported power of the sample in their reports, while we found no explanation about sample size and its power in the VACURG trial. Thus, the VACURG was judged to represent no reliable evidence compared with the other selected trials. Table 2 provides a picture to comparatively observe the methodological quality of selected trials.

**Table 2:** Methodological quality of included trials

***Name***	***SPCG-4***	***PIVOT***	***VACURG***
Randomization allocation (SB)	Adequate	Adequate	Unclear risk
Allocation concealment (SB)	Adequate	Adequate	Unclear risk
Blinding of outcome assessment (SB)	Adequate	Adequate	Unclear risk
Incomplete outcome data (AB)	Adequate	Adequate	Inadequate₳
Selective report(RB)	Adequate	Adequate	Unclear risk
Other bias₢	Adequate	Adequate	Inadequate₳
Sample power	85% power to detect a 25% relative reduction in all-cause mortality	85% power to detect a 25% relative reduction in all-cause mortality	NM
Analysis approach	Intention-to-treatment	Intention-to-treatment	NM
Randomization strategies	A telephone service at office outside the clinical units	Central interactive telephone system	NM
Stratification in randomization	According to degree of differentiation in cancer grade and center	According to sites	NM
Funding source	No industry	No industry	No industry

₰ If no notable bias exist which effect on results of trial/

₳ if there was a great bias which make misleading on results of trial/

₫ if no sufficient data reported to assess the studies/

NM: not mention in trial’s reports/

SB: selection bias/

AB: attrition bias/

RB: reporting bias/

₢ some biases that did not include in the category

To provide meta-analysis of the same outcomes with same time-point, PIVOT and SPCG-4 trials implied the following outcomes: all-cause mortality, prostate-cancer mortality, distant metastases and their subgroup analyses of age at 12 years follow-up; urinary incontinence and erectile dysfunction at 2 years follow-up. Clinical and functional outcomes data of the PIVOT data were extracted from Wlit 2012 (27). Regarding the SPCG-4, we extracted clinical outcomes data from Bill-Axelson 2008 (30) and functional outcomes from Johansson 2009 (34). Individual functional outcomes of SPCG-4 were collected at 2-year follow-up. The VACURG trial was of no sufficient quality to be included in the pooled estimation of the outcomes. Table 3 provides a summary of the following calculations.

**Table 3:** Summaries of pooled effects of treatment in different outcomes

***Outcome***	***Subgroup***	***Studies***	***Statistical method***	***Pooled effect estimate***	***Test of overall effect***	***Heterogeneity I^2^(%)***
All-cause mortality at 12 years	All patients	2	Risk Ratio (IV, Fixed, 95% CI)	0.89 (0.78 to 1.02)	Z= 1.67 P(Z)= 0.1	0
	Age< 65 years	2	Risk Ratio (IV, Random, 95% CI)	0.78 (0.60 to 1.01)	Z= 0.99 P(Z)= 0.32	90
	Age ≥65 years	2	Risk Ratio (IV, Fixed, 95% CI)	0.93 (0.80 to 1.09)	Z=0.92 P(Z)= 0.36	0
prostate-cause mortality at 12 years	All patients	2	Risk Ratio (IV, Fixed, 95% CI)	0.60 (0.38 to 0.94)	Z= 2.22 P(Z)=0.03	0
	Age<65 years	2	Risk Ratio (IV, Fixed, 95% CI)	0.46 (0.27 to 0.76)	Z= 2.99 P(Z)= 0.003	0
	Age ≥65 years	2	Risk Ratio (IV, Fixed, 95% CI)	0.87 (0.56 to 1.34)	Z=0.63 P(Z)= 0.53	0
	PSA>10	2	Risk Ratio (IV, Fixed, 95% CI)	0.61 (0.38 to 0.98)	Z=2.05 P(Z)= 0.04	0
	PSA≤10	2	Risk Ratio (IV, Fixed, 95% CI)	0.77 (0.44 to 1.36)	Z=0.92 P(Z)= 0.36	0
	Gleason<7	2	Risk Ratio (IV, Fixed, 95% CI)	0.61 (0.35 to 1.06)	Z=1.76 P(Z)= 0.08	0
	Gleason≥7	2	Risk Ratio (IV, Fixed, 95% CI)	0.56 (0.34 to 0.92)	Z=2.29 P(Z)= 0.02	0
Distant metastases at 12 years	All patients	2	Risk Ratio (IV, Fixed, 95% CI)	0.55 (0.36 to 0.85)	Z= 2.72 P(Z)= 0.007	44
	Age<65 years	2	Risk Ratio (IV, Fixed, 95% CI)	0.53 (0.36 to 0.78)	Z= 3.18 P(Z)= 0.001	0
	Age ≥65 years	2	Risk Ratio (IV, Random, 95% CI)	0.55 (0.23 to 1.30)	Z= 1.36 P(Z)= 0.17	72
Erectile dysfunction at 2 years	All patients	2	Risk Ratio (M-H, Fixed, 95% CI)	1.88 (1.65 to 2.15)	Z= 9.26 P(Z)<0.00001	0
Urinary incontinence at 2 years	All patients	2	Risk Ratio (M-H, Fixed, 95% CI)	2.95 (1.91 to 4.56)	Z= 4.88 P(Z)<0.00001	0

### Meta-analysis findings All-cause mortality

**Fig. 2:**
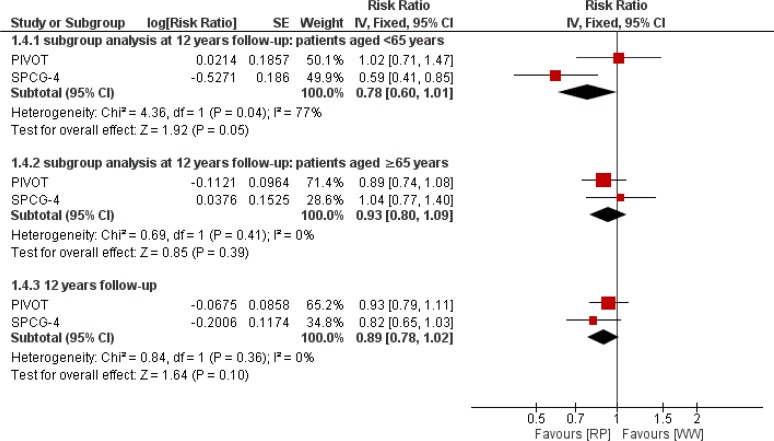
Pooled estimates of all-cause mortality of RP versus WW in patients with localized prostate cancer

Using the Inverse-Variance method, no significant difference was observed between treatments, RR 0.89 [0.78 to 1.02; *P*=0.1 I^2^=0%] (Fig. 2). In subgroup analysis we found no significant effect of the treatments on reducing all-cause death among men of <65 years age, RR 0.78 [0.60 to 1.01] *P*=0.32. I^2^=90%, and ≥65 years with RR 0.93 [0.80 to 1.09] *P*=0.36 I^2^=0%.

### Prostate-cancer mortality

Using Inverse-Variance method, disease-specific death revealed a significant difference between the treatments, RR 0.60 [0.38 to 0.94; *P*=0.03]. Risk difference was −0.036 [−0.066 to −0.007]. There was no heterogeneity between trials (I^2^=0%). Moreover, the number needed to treat to avert one prostate-cause death was 25.

Using Inverse-Variance method, patients of <65 years demonstrated a significant difference favoring RP, RR 0.46 [0.27 to 0.76; *P*=0.003] I^2^= 0%. Risk difference was −0.06 [−0.112 to −0.012]. The number needed to treat to avert one prostate-cause death among younger patients was 16. For patients of ≥65 years, results suggested a similar clinical effect of treatments, RR 0.87 [0.56 to 1.34; *P*=0.53]. Additionally, there was no heterogeneity between trials (I^2^= 0%).

Disease-specific death in patients with PSA>10 was significantly lower in RP group that WW group, RR 0.61 [0.38 to 0.98; *P*=0.04]. There was no heterogeneity between trials (I^2^= 0%). But in patients with PSA≤10 number of disease-specific death between two groups was not significant, RR 0.77 [0.44 to 1.34; *P*=0.36]. There was no heterogeneity between trials (I^2^= 0%).

**Fig. 3:**
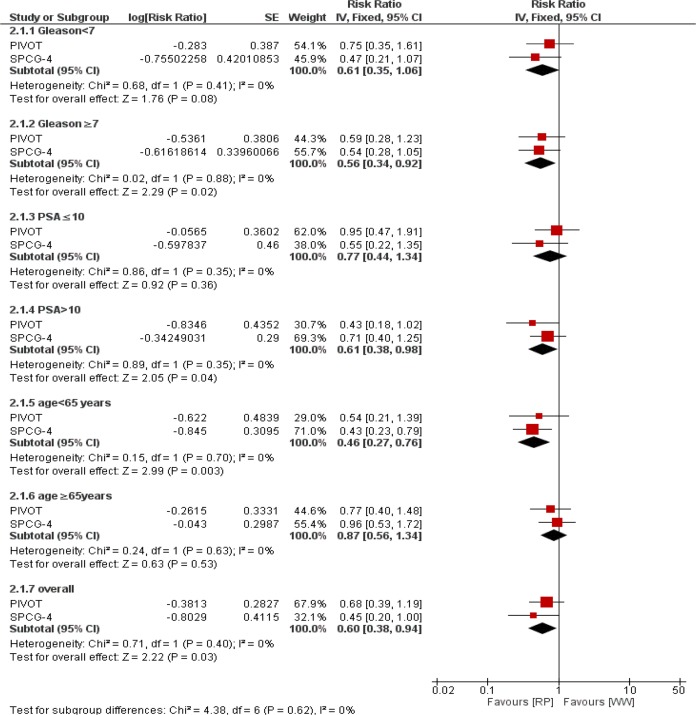
Pooled estimates of prostate-specific mortality of RP versus WW in patients with localized prostate cancer

Disease-specific death in patients with Gleason≥7 was significantly lower in RP group that WW group, RR 0.56 [0.34 to 0.92; *P*=0.02]. There was no heterogeneity between trials (I^2^=0%). But in patients with Gleason<7 number of disease-specific death between two groups was not significant, RR 0.61 [0.35 to 1.06; *P*=0.08]. There was no heterogeneity between trials (I^2^=0%). Figure 3 represents the forest plot of prostate-cancer death and its subgroup results.

### Distant metastases

**Fig. 4:**
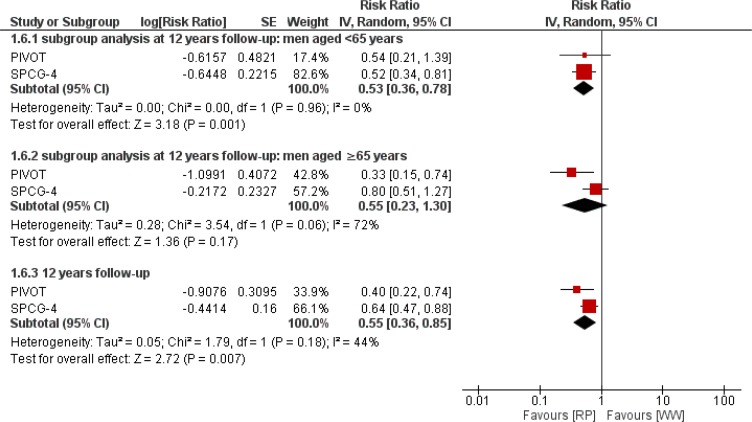
Pooled estimates of distant metastases outcome of RP versus WW in patients with localized prostate cancer

Using inverse-variance method, distant metastases rate showed significant difference favoring RP, RR 0.55 [0.36 to 0.85; *P*=0.006] I^2^= 44% risk difference −0.060 [−0.092 to −0.028]. The number needed to treat to avert one distant metastasis was 16. In patients with <65 years, it demonstrated significant lower rate favoring RP, RR 0.53 [0.36 to 0.78; *P*=0.001] I^2^= 0% risk difference −0.059 [−0.115 to −0.004]. The number needed to treat to avert one distant metastasis among younger patients was 16. But we found no significant difference between treatments among men aged ≥65 years, RR 0.55 [0.23 to 1.30; *P*=0.17] I^−2^= 72%. Figure 4 shows forest plot of distant metastases and its subgroups.

### Urinary incontinence

**Fig. 5:**
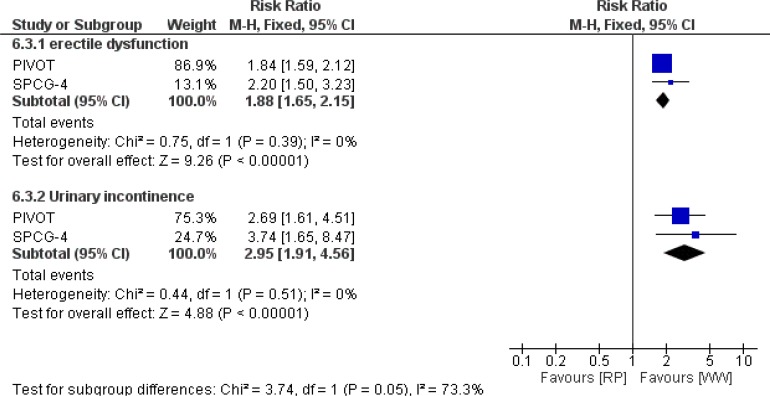
Pooled estimates of urinary incontinency and erectile dysfunction outcomes of RP versus WW in patients with localized prostate cancer

We applied Mantel-Haenszel method and found significant difference among participants in favor of WW group at 2 years follow-up, RR 2.95 [1.91 to 4.56 *P*<0.00001] risk difference −0.138 [−.189 to −0.088]. The number needed to treat to avert one the outcome was 7. There was no heterogeneity between trials (I^2^= 0%). The Fig. 5 illustrates the results.

### Erectile dysfunction

Our result represented significant difference among participants in favor of WW group at 2 years follow-up, RR 1.88 [1.65 to 2.15; *P*<0.00001] risk difference −0.378 [−0.446 to −0.310]. The number needed to treat to avert one erectile dysfunction was 2. Mantel-Haenszel method was used. There was no heterogeneity between trials (I^2^= 0%). The Fig. 5 illustrates the forest plot of result of functional outcomes.

## Discussion

### Main findings

Although non-significant effect of Radical prostatectomy was observed at 12 years follow-up on reducing all-cause mortality, we found a significant difference between treatments on prostate-cause mortality and distant metastases favoring radical prostatectomy at 12 years follow-up. In subgroup analysis of age, we found that, radical prostatectomy, in younger patients<65 years had significant effect on prostate-cause mortality and distant metastases rate while, there was no evidence in favor of a specific treatment for older patients, ≥65 years. We found significant effect of urinary incontinence and erectile dysfunction among participants opted for WW strategy during 2 years.

This is likely the first study which reports pooled clinical and functional results of recent randomized controlled trials comparing RP with WW strategy in treatment of clinically localized prostate cancer patients. We found three trials with 1568 participants. Moreover, included trials in meta-analysis would have adequate methodological quality. We demonstrated how age could play substantial role in mortality rate of patients who underwent surgery. Non-significant effect of RP on reducing all-cause death among overall patients was observed. However, future reports of ongoing trials (21) and exploring the outcomes in broader time-point are required in this regard. In subgroup analysis of age<65 years, we even proved RP had significant effect on reducing disease-specific death and distant metastases, by including ongoing trial’s findings. Functional outcomes more than two items which pooled in this review should be analyzed and great attention ought to be paid to psychological status and health-related quality of life. Exploring impact of Gleason score and PSA level on clinical outcomes so as to manage clinically localized prostate cancer is useful; but SPCG-4 trial found no representative categories to explore role of these criteria. Race had impact on progression and dying from prostate cancer (37–39). One third of the participants in PIVOT were African-American increased risk of dying from prostate cancer. Paucity of data on diversities of ethnicity in SPCG-4 trial prevented us from analyzing disease-specific death among different races. Furthermore, included trials had different method regarding detection of patients. Majority of participants in PIVOT trial were detected through PSA screening (76%), while few participants in SPCG-4 trial were detected with PSA level (5%); and, most of them had clinically detected prostate cancer. This diversity is important when some studies highlighted that lead time bias caused by PSA screening (40–42) may influence the time of occurrence of disease-specific death and local progression.

## Conclusion

We found some evidence to recommend RP as a primary treatment for younger patients who suffered from clinically localized prostate cancer at a 12-year follow-up. However, including reports of ongoing trials and analyzing in broader time-point may be allowed to precisely analyze clinical and functional outcomes. We found better sexual and urinary functions among patients who received WW strategy during a 2-year follow-up. However, other variables such as psychological status, health-related quality of life, race differences and PSA screening should be substantiated to make better decision about these patients.

## Ethical considerations

Ethical issues (Including plagiarism, informed consent, misconduct, data fabrication and/or falsification, double publication and/or submission, redundancy, etc.) have been completely observed by the authors.
